# Fibromyalgia and Skin Disorders: A Systematic Review

**DOI:** 10.3390/jcm13154404

**Published:** 2024-07-27

**Authors:** Martina D’Onghia, Jacopo Ciaffi, Laura Calabrese, Linda Tognetti, Elisa Cinotti, Pietro Rubegni, Bruno Frediani, Francesco Ursini

**Affiliations:** 1 Department of Medical, Surgical and Neurological Sciences, Dermatology Section, University of Siena, 53100 Siena, Italy; martina.donghia@gmail.com (M.D.); laura.calabrese@unisi.it (L.C.); elisa.cinotti@unisi.it (E.C.); pietro.rubegni@unisi.it (P.R.); 2 Medicine and Rheumatology Unit, IRCCS Istituto Ortopedico Rizzoli, 40136 Bologna, Italy; francesco.ursini2@unibo.it; 3 Department of Biomedical and Neuromotor Sciences (DIBINEM), University of Bologna, 40123 Bologna, Italy; 4 Rheumatology Unit, Department of Medicine, Surgery and Neurosciences, University of Siena, 53100 Siena, Italy; bruno.frediani@unisi.it

**Keywords:** fibromyalgia, skin disorders, psoriasis, urticaria, vitiligo, hidradenitis suppurativa

## Abstract

**Background:** Fibromyalgia is a complex multifaceted syndrome primarily characterised by chronic musculoskeletal pain, fatigue, and functional symptoms. Although FM is known to be associated with several comorbidities, the aim of this systematic review was to comprehensively examine the available evidence regarding the relationship between FM and dermatological manifestations. **Methods:** We followed the Preferred Reporting Items for Systematic Reviews and Meta-Analyses (PRISMA) guidelines, and MedLine and Web of Science (WOS) databases were searched up to June 2023. After removing duplicate records, 21 articles were deemed eligible for inclusion in the qualitative synthesis. **Results:** Overall, the included studies revealed an increased frequency of FM among patients with cutaneous diseases, including psoriasis, chronic urticaria, contact allergy, acneiform disorders, hidradenitis suppurativa, and vitiligo. Additionally, the presence of comorbid FM may intensify skin conditions, which has a negative impact on quality of life and vice versa. **Conclusions:** Although the causal mechanisms of FM are still far from being understood, this systematic review suggests a relationship between FM and skin disorders. However, further research is encouraged in this area.

## 1. Introduction

Fibromyalgia (FM) is a common condition primarily characterised by chronic widespread pain, fatigue, sleep disturbances, and cognitive disorders [1]. Additional symptoms commonly include irritable bowel syndrome, headache, paraesthesia, and psychological comorbidities, such as depression or anxiety; recent data supported also a possible role of obesity [2]. FM is the third most frequent musculoskeletal condition, and its prevalence varies between 1.7% and 6% worldwide [3]. On these premises, the significant impact of FM on patients’ quality of life (QoL), and the resulting healthcare costs, is not surprising [4]. 

The pathogenesis of FM is poorly understood [5]. Globally, it is considered to result from a complex interplay between the dysfunction of pain control mechanisms and immune, endocrine, genetic, and psychosocial factors [3]. However, the real nature of FM remains puzzling, and a considerable proportion of physicians still fail to recognise this syndrome. 

Notably, although several comorbidities have been associated with FM development, including inflammatory rheumatic diseases [6], this condition has not been commonly linked to cutaneous manifestations. 

Previous studies have outlined objective differences between skin biopsies from patients with FM and healthy people [7], showing high mast cell counts and expression of inflammatory cytokines [8,9], alteration in collagen metabolism [10], cutaneous microcirculatory [8], autonomic nervous system dysfunction, and high cutaneous opioid receptors in patients with FM [11]. 

In this context, no study has systematically reviewed the possible links between FM and skin manifestations. Thus, we conducted a systematic review to shed light on the relationship between FM and cutaneous findings, including epidemiological associations and reciprocal interactions. 

## 2. Materials and Methods

### 2.1. Search Strategy

MedLine (via PubMed) and Web of Science (WOS) databases were searched up to 4 June 2023. The main search in MedLine was performed using the string “(fibromyalgia OR “chronic fatigue syndrome” OR “chronic fatigue disorder”) AND (psoriasis OR sweating OR hyperhidrosis OR dermatitis OR pruritus OR itch OR prurigo OR raynaud OR acne OR rosacea OR burning OR folliculitis OR urticaria OR “hair loss” OR trichotillomania OR rash OR eczema OR ulcers OR lichen OR vasculitis OR “Hidradenitis suppurativa” OR “Livedo reticularis” OR morphea OR mastocytosis OR “skin disease” OR “cutaneous symptoms” OR dermatologic* OR eczema OR erythema OR vitiligo OR keratosis OR onychophagia)”. 

The primary search in WOS was “[TS = ((fibromyalgia OR “chronic fatigue syndrome” OR “chronic fatigue disorder”) AND (psoriasis OR sweating OR hyperhidrosis OR dermatitis OR pruritus OR itch OR prurigo OR raynaud OR acne OR rosacea OR burning OR folliculitis OR urticaria OR “hair loss” OR trichotillomania OR rash OR eczema OR ulcers OR lichen OR vasculitis OR “Hidradenitis suppurativa” OR “Livedo reticularis” OR morphea OR mastocytosis OR “skin disease” OR “cutaneous symptoms” OR dermatologic* OR eczema OR erythema OR vitiligo OR keratosis OR onychophagia)). 

**Figure 1:**
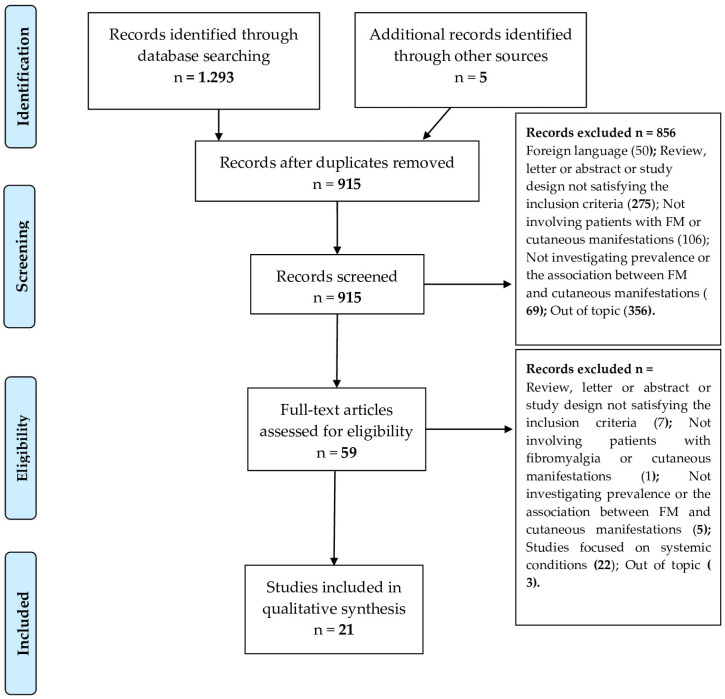
PRISMA–compliant study selection flow chart [13].

In addition, relevant keywords were combined for a manual search, and the bibliographies of selected articles were examined. The inclusion of the term “chronic fatigue syndrome” as a keyword was strategically chosen to enhance the search sensitivity. This was performed to identify studies on chronic fatigue syndrome (CFS) that also included participants meeting the criteria for Fibromyalgia (FM), acknowledging the clinical similarities between the two conditions, despite the primary focus on FM in our article. The search strategy was developed and executed by one of the authors (MD), with oversight from a senior investigator (FU). There were no restrictions based on the date of publication. Our manuscript was prepared in accordance with the Preferred Reporting Items for Systematic Reviews and Meta-Analyses (PRISMA) guidelines (Figure 1) [12].

### 2.2. Eligibility Criteria

For this review, only English-language articles were considered eligible for consideration. Studies must be published as full-text original articles in international, peer-reviewed journals for inclusion in the final review. Eligible study designs included randomised controlled trials (RCTs), quasi-randomised controlled trials (where treatment allocation was determined by methods such as alternation, use of alternate medical records, date of birth, or other non-random methods), prospective or retrospective cohort studies, and cross-sectional studies. All studies investigating the direct relationship between FM and skin disorders were considered relevant for inclusion in the qualitative synthesis.

### 2.3. Study Selection Process and Data Extraction

For the first-step evaluation, two investigators (FU and MD) independently reviewed the abstracts and titles after removing duplicate records. Then, the full texts of the remaining papers were independently evaluated by the same two reviewers to determine their inclusion in the final review. Until a final consensus was reached, disagreements among the reviewers were settled by discussion with a third investigator (JC). A flowchart of the selection process is presented in Figure 1. Further studies were identified through a bibliography of relevant articles. 

### 2.4. Quality Assessment

The Newcastle-Ottawa Quality Assessment Scale (NOS) [14], a widely used tool for assessing bias risk in cohort and cross-sectional studies, was adopted to evaluate the quality of the included studies (Table 1). 

## 3. Results 

### 3.1. Results of Systematic Investigation

Overall, 677 PubMed and 616 WOS records were identified. A manual search identified five additional studies. After eliminating duplicates, a total of 915 studies were reviewed. After screening the titles and abstracts, 856 were eliminated, and the full texts of 59 articles were evaluated. In total, 21 articles were considered suitable for inclusion in the qualitative synthesis (Table 2). The NOS scale was used to perform a quality assessment, which revealed a high risk of bias. None of the included studies reached a score higher than 7, which was considered high quality (Table 1).

### 3.2. Psoriasis 

Five studies have focused on the relationship between psoriasis (PsO) and FM [19,23,24,25,32] (Table 2). Two papers investigated the frequency of FM and its effect on PsO outcomes [25,32]. Thune et al. [32] examined 1269 consecutive patients with PsO in a cross-sectional study to determine the effects of concomitant FM. Of these, 105 (8.3%, 97.6% females) met the 1990 FM criteria set forth by the American College of Rheumatology (ACR). Notably, compared with the non-FM group, the FM + PsO patients had higher tender point count (TPC), visual analogue scale (VAS) pain score, prevalence of sleep disruptions, morning soreness, and daytime weariness (all *p* < 0.05). Similarly, Mathkhor et al. [25] conducted a cross-sectional study and found a 30% prevalence of FM in 70 patients with PsO, who also showed higher psoriasis area severity index (PASI) scores than those without FM (57.9 ± 4.6 vs. 15.5 ± 4.6 respectively, *p* < 0.05). 

On the other hand, three authors have explored the prevalence of PsO in patients with FM [16,17,18]. In a cross-sectional study of 825 patients with FM by Laniosz et al. [24], a 2.2% overall prevalence of PsO was reported, which appears to be similar to the prevalence observed in the general population of Western countries [36]. Erdogan et al. [19] similarly showed that PsO may not be overrepresented in the FM population (*p* = 0.498). Conversely, a population-based study by Kridin et al. [23] using a large database of 18,598 patients with FM and age- and gender-matched controls without FM (n = 36,985) revealed that PsO prevalence was higher in patients with FM than in controls (6.7% vs. 4.8%, respectively; OR 1.4; 95% CI: 1.3–1.5, *p* < 0.001). 

### 3.3. Chronic Urticaria

Four studies evaluated the association between FM and chronic urticaria (CU) [26,28,33,35]. In the first study published by Oktayoglu et al. [28], 40 patients with CU and 38 healthy controls were enrolled. Interestingly, the frequency of FM was higher among patients with CU than controls (32.5% vs. 10.5%, *p* = 0.019). Moreover, patients with CU and concomitant FM had worse quality of life (QoL) total scores as assessed by the Nottingham Health Profile (NHP) scores than those without FM (*p* < 0.001); however, the depression and anxiety scores were not significantly different (*p* = 0.070 and *p* = 0.069 respectively).

Similarly, Torresani et al. [33] evaluated 126 patients with CU, among whom a surprisingly high proportion (70.6%) satisfied the FM criteria. Notably, the 89 patients with FM in the CU group comprised 89 females and 21 males, and only 15 of them were aware of having FM. 

Recently, Mathkhor et al. [26] conducted a study on 82 patients with CU and 86 healthy controls and reported that 60.9% of them suffered from widespread pain, whereas 28 patients met the ACR 1990 FM criteria (34.1%, 71.4% females). Furthermore, the authors demonstrated that FM-associated clinical features, such as morning stiffness, sleep disturbances, fatigue, anxiety, and depression, were more frequent in patients with CU than in controls (all *p* < 0.05). Additionally, they found that Autologous Serum Skin Test (ASST) and severe urticarial total severity score (TSS) were more frequent in patients with CU having FM than in those without FM (*p* < 0.05).

On the other hand, Yener et al. [35] compared 72 patients with idiopathic CU with 67 sex- and age-matched healthy controls and showed that the frequency of FM was similar between the two groups (9.7% vs. 4.5%, *p* = 0.32). However, the authors found that FM symptom duration, TPC, and FIQ scores were higher in patients with idiopathic CU than in controls (all *p* < 0.05). In addition, patients with idiopathic CU had significantly higher VAS scores (*p* = 0.01); further, the urticaria activity score (UAS) was correlated with the presence of FM, symptom duration of FM, tender point number, and FIQ and VAS scores (all *p* = 0.0001). Finally, logistic regression analysis revealed that UAS was an independent predictor of the presence of FM (b = 0.34, *p* = 0.003).

### 3.4. Contact Allergy

Two studies investigated contact allergy rates in individuals with FM [17,20]. First, Bruze et al. [17] performed a baseline patch test series and an extended dental series in 120 individuals with FM and two control groups, consisting of patients with contact dermatitis and individuals from the general population. Overall, 44.5% of patients with FM had at least one contact allergy. However, the comparison between the FM group vs. the dermatitis group showed no difference in the number of individuals with at least one allergy or the total number of positive reactions (*p* = 0.13). On the other hand, when the FM group was compared with the general population, a higher number of positive reactions was observed in the FM group (*p* < 0.0001). Specifically, higher rates in the FM group were detected for nickel (*p* < 0.0004), Myroxolon pereirae (*p* < 0.00004), and fragrance mix I (*p* = 0.011).

In a similar study from the same author group [20], there were significantly more individuals with at least one allergy to acrylates, methacrylates, and substances used in the acrylate polymerisation process in patients with FM compared with patients with oral symptoms suspected of having contact allergy (*p* = 0.016), whereas allergy to gold sodium thiosulphate (GSTS) was significantly higher in the FM group compared with patients with contact dermatitis (*p* = 0.043).

### 3.5. Acneiform Disorders

Three studies have investigated the association between acneiform disorders and FM [15,27,34]. Acar et al. [15] explored the relationship between rosacea and FM in a cross-sectional study that included 100 female patients with rosacea and 100 age- and sex-matched controls. The frequency of FM was significantly higher in the rosacea group than in the control group (37% vs. 21%; *p* = 0.019). The mean duration of FM in the rosacea group was significantly higher than in the control group (*p* = 0.001), while, in 64.8% of rosacea cases, FM started before the occurrence of skin symptoms. No significant differences were found in terms of age at onset of FM, FIQ, and VAS scores between the two groups (*p* > 0.05); Dermatology Life Quality Index (DLQI) scores were correlated with FIQ scores in patients with rosacea (r = 0.39; *p* = 0.017). 

Another study by Yazmalar et al. [34] compared 88 patients with acne vulgaris with 76 age- and sex-matched controls and showed that the frequency of FM was significantly higher in patients with acne (21.6% vs. 5.3%, *p* < 0.05). A comparison of patients with and without FM indicated no significant differences in age, global acne score, and morning stiffness (all *p* > 0.05). Furthermore, no significant correlation was found between the severity of acne and the VAS-pain score (r = −0.107, *p* = 0.320), number of tender points (r = −0.194, *p* = 0.070), disease duration (r = −0.074, *p* = 0.491), or anxiety score (r = −0.075, *p* = 0.490). Moreover, patients with FM had higher rates of arthralgia, headache, irritable bowel syndrome, fatigue, anxiety, sleep disturbance, and number of tender points (all *p* < 0.05) than those without FM.

Similarly, Mathkhor et al. [27] investigated the prevalence of FM and its associated symptoms among 91 patients with acne vulgaris and 84 sex- and age-matched controls. Overall, 23.0% of patients fulfilled the 1990 ACR FM criteria, compared with 1.2% of the control group (*p* < 0.05). In addition, the frequency of widespread pain and FM-associated features, including sleep disturbances, anxiety, depression, fatigue, and irritable bowel syndrome, were more frequent in the inpatient group than in the controls, reaching a statistically significant difference (all *p* < 0.05). Finally, global acne score (34.8 ± 2.3 vs. 22.17 ± 3.3, *p* < 0.001) and body mass index (BMI) (28.8 ± 1.8 kg/m^2^ vs. 20.6 ± 1.8 kg/m^2^, *p* < 0.001) were higher in acne patients with FM.

### 3.6. Hidradenitis Suppurativa

Prens et al. [30] investigated the prevalence of hidradenitis suppurativa (HS) in a large population-based cohort in the northern Netherlands with the aim of identifying potentially associated comorbidities of HS through a national web-based survey. Among the 56,084 respondents, of which 6156 had HS (2.1%), a significant association with FM (OR 2.26, 95% CI: 1.64–3.11) and chronic fatigue syndrome (OR 1.72, 95% CI: 1.06–2.78) was identified. 

### 3.7. Vitiligo

Two of the studies investigated the association between vitiligo and FM [16,21]. Askin et al. [16] compared 35 patients with generalised vitiligo with 45 sex- and age-matched controls and found that the frequency of FM was significantly higher in patients with vitiligo (34.3% vs. 11.1%, *p* = 0.015). Interestingly, the authors found that vitiligo patients with concomitant FM not only had worse scores on the FIQ and measures of physical functioning, psychological status, fatigue, stiffness, depression, anxiety, and sleep quality; but that the presence of coexisting FM increased the severity of vitiligo itself, as measured by the Vitiligo Area Scoring Index (VASI). 

Consistent with this, Khudhair et al. [21] confirmed that a higher proportion of patients with vitiligo (12%) had a diagnosis of FM compared with controls (7%). Because there were no male patients with vitiligo and FM, the authors performed a sex-stratified analysis, revealing a significantly higher prevalence rate of FM among female patients with vitiligo (22.2%) than in controls (9.5%). Overall, the risk of FM was increased by 2.71 times in patients with vitiligo compared with controls (OR = 2.71, 95% CI: 1.13–6.51, *p* = 0.022). Moreover, having a severe form of vitiligo conferred a 15-fold higher risk of FM compared with a mild to moderate condition (OR = 15.0, 95% CI: 3.2–69.8, *p* < 0.001). Finally, a longer duration (10+ years) of vitiligo was associated with a higher prevalence rate of FM (30.4% vs. 14.3%). 

### 3.8. Miscellaneous

Six authors have focused on different dermatological findings in FM [18,19,22,24,29,31]. First, Dogramaci et al. [18] aimed to highlight the possible association between FM and dermatological diseases in a cross-sectional study of 66 female patients diagnosed with FM. Their analysis revealed that the incidence of xerosis (respectively 32 vs. 13; *p* < 0.001) and neurotic excoriation (11 vs. 0 cases, *p* < 0.001) were significantly higher in the FM group than in the control group. In addition, the presence of skin disorders in patients with FM was not associated with a statistically significant impact on SF-36 quality of life (*p* > 0.05). 

Laniosz et al. [24] evaluated different dermatological domains in a cohort of 845 patients with a confirmed diagnosis of FM. The authors found a subjective increase in sweating in 270 (32%) subjects, followed by a burning sensation of the skin or mucous membranes in 29 (3.4%), and various unusual cutaneous sensations in 14 (1.7%). Pruritus without an identified cause was reported by 28 patients (3.3%), with another 16 patients (1.9%) showing neurotic excoriations, prurigo nodules, or lichen simplex chronicus. Finally, some form of dermatitis other than neurodermatitis was found in 77 patients (9.1%). 

In 2016, Erdogan et al. [19] published the results of a cross-sectional study on 105 female patients with FM and 105 healthy controls. They observed that the percentage of patients with at least one cutaneous symptom in the FM cohort was higher than that in the control group (92.4% vs. 42.9%; *p* < 0.001). Additionally, the rate of skin manifestations such as pruritus (69.5% (*p* < 0.001), burning (49.5% (*p* < 0.001), hyperhidrosis (67.6% (*p* < 0.001), and numbness and tingling (34.3%) were more common in the FM group than in the control group (*p* < 0.001). The most common dermatologic disorders in patients with FM were xerosis (44.7%), lichen simplex chronicus (15.2%), acne (10.4%), contact dermatitis (8.5%), neurotic excoriation (6.6%), tinea pedis (6.6%), melasma (4.7%), and seborrhoeic dermatitis (3.8%). Moreover, a significant statistical difference was found between the two groups in terms of xerosis (*p* = 0.002), lichen simplex chronicus (*p* = 0.011), neurotic excoriation (*p* = 0.031), tinea pedis (*p* = 0.007), and seborrhoeic dermatitis (*p* = 0.043). However, dermatologic disorders or cutaneous symptoms in patients with FM did not reflect significant differences in SF-36 physical function, physical role, pain, general health, vitality, social function, emotional role, and mental health scores (*p* > 0.05).

Interestingly, Kepekçi et al. [22] investigated whether there was a difference between male and female patients with FM and matched male and female healthy volunteers concerning dermatological symptoms. Overall, the main finding was that the incidence of lichen simplex chronicus was significantly higher in female patients with FM than in healthy female participants (*p* = 0.006). 

Furthermore, in a cross-sectional study on 77 FM patients and 74 controls, Surucu et al. [31] found that 37.1% of patients with FM patients vs. 19.9% of healthy individuals had at least one cutaneous symptom (*p* < 0.01). Moreover, the comparison of cutaneous symptoms between the FM and control groups showed a significant difference in hyperidrosis, burning, and lichen simplex chronicus (all *p* < 0.01), while no differences were observed in terms of acne, urticaria, or seborrhoeic dermatitis (all *p* > 0.05). Lastly, the SF-36 total score (*p* = 0.02) and physical role (*p* < 0.01) were found to be significantly lower in patients with FM who had dermatological symptoms. 

To conclude, Oktayoğlu et al. [29] focused on exploring the presence of pathergy, a non-specific hypersensitivity reaction to minor trauma, in 46 patients with FM and 28 individuals with Behcet disease, concluding that there was no evidence of pathergy in the FM group.

## 4. Discussion

Fibromyalgia is a multifaceted disease that has a major impact on individuals and society [1]. Although several comorbidities have been associated with FM, such as psychological diseases [37] and inflammatory rheumatic conditions [6], little is known regarding their potential association with cutaneous disorders. On this background, the aim of our systematic review was to identify all available clinical evidence on this topic. 

**Figure 2:**
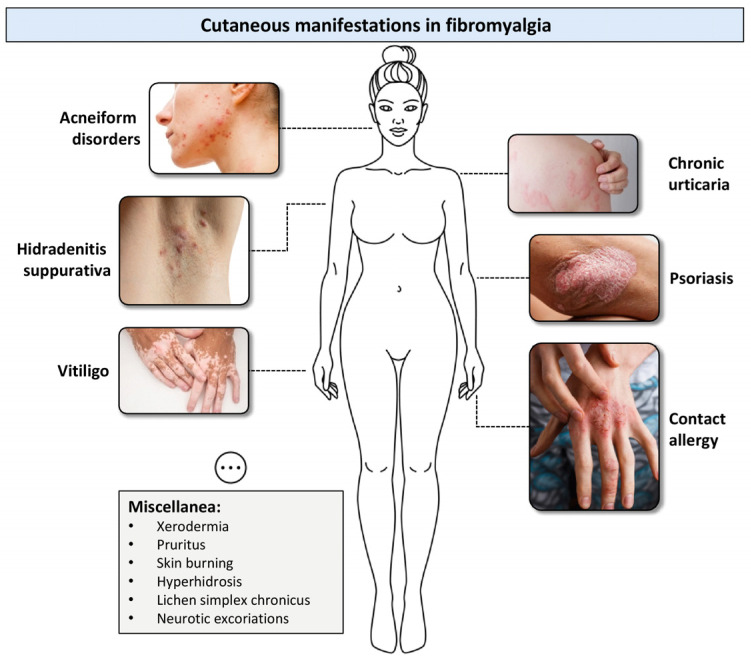
Skin disorders associated with Fibromyalgia.

The studies retrieved in this systematic review suggested a potential link between FM syndrome and skin conditions (Figure 2). Even if a clear causal relationship cannot be ascertained, speculative pathophysiological hypotheses based on the current literature may include dysregulation of inflammatory and immunological pathways, an altered autonomic nervous system, and psychosocial distress. 

Along with genetic, environmental, and neurohormonal factors, research to date has confirmed the contribution of inflammation to the pathogenesis of FM. A growing body of evidence has shown elevated concentrations of pro-inflammatory cytokines released from immune cells (monocytes, T cells, and macrophages) in the serum/plasma of patients with FM, including IL-6, IL-8, IL-1β, TNF, and IL-10, as well as chemokine, lipid mediators, oxidative stress markers, and plasma-derived factors [38]. Additionally, animal studies have provided strong support for the role of cytokines and chemokine in enhancing pain perception [39], central sensitisation [40], and the development of chronic muscle pain [41]. Furthermore, pro-inflammatory cytokines have been suggested to regulate other FM symptoms, such as depression [42], fatigue [43], and sleep disturbances [44], supporting the potential role of inflammatory mechanisms in the broad range of FM clinical symptoms. It is well-established that inflammation plays a significant role in several dermatological conditions, including acne [45], rosacea [46], PsO [47], HS [48], and CU [49]. Therefore, even though inflammation may not be the main contributor to the pathophysiology of FM, inflammatory skin diseases may contribute to or reinforce the inflammatory state in patients with FM. This, in turn, can amplify and sustain nociceptive signals from peripheral areas and elicit pain perception inducing central sensitisation [50].

In particular, five studies have focused on the relationship between FM and PsO [19,23,24,25,32]. Two authors suggested that patients with psoria had a higher prevalence of FM (8–30%), with a very high impact on the symptoms of PsO [25,32]. On the other hand, patients with FM had a slightly higher prevalence of PsO (2.2–6.7%) than the control group [19,23,24]. On this basis, a possible hypothesis beyond this bidirectional relationship may be represented by common inflammatory pathogenetic mechanisms, as suggested by the increased levels of interleukin 1 beta, IL-6, IL-8, and tumour necrosis factor (TNF)-α observed in both conditions [51,52]. Such an explanation could also underlie the relationship between FM and HS, a chronic inflammatory skin disease primarily affecting apocrine gland-rich areas of the body, as demonstrated by Prens et al. [30]. However, further investigations are required in this field. 

Mast cells have been identified as possible contributors to the inflammation observed in FM. Notably, the levels of IL-6 and TNF-alpha, two primary pro-inflammatory cytokines released by mast cells, are higher in patients with FM than in healthy controls [53]. Additionally, an increased number of mast cells, as well as increased mast cell degranulation, have been detected in skin biopsies of patients with FM, although their role in FM pathogenesis remains unclear [8,54]. Mast cells are intimately linked to the pathogenesis of CU and other allergic disorders [55,56]. Intriguingly, Tuncer et al. found an allergy background in patients with FM [57]. From our review, four studies explored the relationship between FM and CU. Although three authors demonstrated that FM was overrepresented among patients with CU [26,28], a study by Yener et al. [35] failed to support these hypotheses. Furthermore, two studies on patients with FM demonstrated an increased rate of contact allergy, a common inflammatory skin disorder characterized by erythematous pruritic rash [17,20]. Although the precise putative mechanism behind this relationship is far from being clarified, it is possible to hypnotize that FM patients may have expressed a yet unidentified risk factor that increases the likelihood of developing contact allergy (“sensitisation hypothesis”), or that exposure to certain sensitisers is instrumental for the development of FM (“elicitation hypothesis”). In this regard, the oral mucous membranes may be a potential route of sensitisation in individuals with FM. 

Higher serum levels of neuropeptides, such as neuropeptide Y, corticotropin-releasing hormone, substance P, and its structurally related hemokinin-1, have been observed in patients with FM [3], but elevated levels of substance P have also been detected in skin conditions such as PsO or CU [55,58]. These peptides can, in turn, promote mast cell secretion of pro-inflammatory cytokines, which, in a vicious circle, can further stimulate neurones to release more neuropeptides [1]. In the skin, intensive and bidirectional crosstalk between the nervous and immune systems may be promoted by the proximity between immune cells in the skin barrier and peripheral nerves, which represents another possible link between FM and skin disorders. 

According to the current literature, dysregulation of the autonomic nervous system has been documented in patients with FM, with the sympathetic autonomic nervous system exhibiting both hyperactivity and hyporeactivity [59]. Dysautonomia, or altered activation of the autonomic nervous system, has been proposed as a potential explanation for various complaints associated with FM, including hyperhidrosis, as observed in our review by three authors [19,24,31]. Additionally, given that blood vessels are regulated by sympathetic, parasympathetic, and sensory nerves, dysfunction in these mechanisms could lead to increased skin blood flow, angiogenesis, and vasodilation. These physiological changes are characteristic features of dermatological conditions like acne and rosacea, which are marked by facial erythema, flushing, and oedema [46,60]. Accordingly, the results of our review support an association between acneiform disorders and FM [27,34]. Not only were the prevalence and mean duration of FM significantly higher in the rosacea patients than in the control group (*p* = 0.019 and *p* = 0.001, respectively), but DLQI scores were also correlated with FIQ scores in patients with rosacea (r = 0.39; *p* = 0.017).

The fundamental and debilitating symptom of FM is chronic widespread pain. Although the pathophysiology of FM is challenging, pain centralization has emerged as the main hypothesis. The central nervous system can modulate a complex network of interactions with the peripheral region, leading to the augmentation of pain perception [1]. Chronic pain often mirrors pruritus, another major medical condition. Pruritus is one of the most common dermatologic complaints, and it can be alone or associated with other skin disorders, such as PsO, atopic dermatitis, and others [61]. Chronic pain and pruritus are encoded by common mechanisms, including peripheral and central sensitisation [62], loss of inhibition in the spinal cord, and neuroimmune and neuroglial interactions [63]. In our review, only one study demonstrated a higher percentage of pruritus in patients with FM (69.5%) compared with healthy controls (*p* < 0.001), while a high percentage of burning [19,24] and neurotic excoriation [31] was assessed in patients with FM. The increased number of opioid receptors, in addition to increased numbers of inflammatory cytokines and mast cells, have been shown to further contribute to pruritus, burning sensation, or other neuropathic discomfort that is common in patients with FM [64]. Indeed, increased levels of μ, κ, and δ opioid receptors were observed in FM skin compared with healthy controls [65]. However, there is a lack of literature on this topic, and we believe that further studies are required to shed light on this association. Notably, the neuropathic symptoms in patients with FM could also reflect underlying small-fibre neuropathy, since evidence of small-fibre dysfunction has been reported in nearly 50% of FM cases [66]. 

Other possible puzzle pieces can be found in the literature because FM has a clear association with several autoimmune diseases [67]. In our analysis, two studies demonstrated a relationship between FM and vitiligo [16,21], an autoimmune disease of the skin that targets pigment-producing melanocytes and results in patches of depigmentation [68]. 

Another explanation beyond the link between FM and skin manifestations could be the role of psychological distress. Over the years, it has been suggested that somatisation of psychological distress could contribute to the onset or exacerbation of FM symptoms [69,70]. In support of this hypothesis, population-based studies have demonstrated that psychological distress, particularly early-life trauma such as parental loss and abuse, can predict the development of chronic widespread pain and FM [71]. Similarly, it is well known that stressors can directly affect, reveal, or even exacerbate a great number of skin conditions, including dermatitis, acne, vitiligo, rosacea, PsO, and CU [72]. There is increasing evidence that stress may modulate the hypothalamic–pituitary–adrenal axis and contribute to a general inflammation state through the release of neuropeptides, neurotrophin, lymphokines, and other chemical mediators from nerve endings and dermal mast cells [73]. Although several studies have shown that hypothalamic–pituitary–adrenal axis abnormalities exist in FM, chronic fatigue syndrome (CFS), and other stress-related disorders [74], skin reactivity to various stressors might be due to dermal mast cells, as they show close connections with sensory nerve endings and may release a large number of proinflammatory mediators [75]. Interestingly, the expression of adhesion molecules appears to be modulable by stress-related pathways. In addition, L-selectin and b2-integrin expression on the surface of polymorphonuclear leukocytes was considerably reduced in patients with FM, according to Kaufmann et al. [76]. In particular, such adhesion molecules play a role in eliminating infectious organisms and removing toxic substances and debris from the body. Therefore, we might hypothesise a possible role in skin conditions like acne or rosacea, where cutaneous infections represent significant triggers [77]. Additionally, a higher expression of genes related to corticotrophin-releasing hormone (released in response to physiologic stress) than in normal skin has been observed in patients with acne vulgaris [78,79]. Finally, FM and dermatological conditions are associated with an increased risk of psychiatric disabilities. The bidirectional association between depression and anxiety has been widely reported in dermatological conditions, including vitiligo, acne, rosacea, and CU [80,81,82,83]. Underlying psychiatric comorbidity is estimated to occur in up to one-third of dermatologic patients, and psychiatric illness may either be the cause or consequence of dermatologic disease [84]. Intriguingly, a recent hypothesis evoked the dysregulation of common inflammatory and immunological pathways, such as neurochemical dysfunction, serotonergic system hypofunction, and changes in hypothalamic–pituitary–adrenal axis activity, in psychiatric and dermatologic disorders [85]. A close link between FM and psychiatric comorbidities has also been described in the literature [20]. On this basis, since FM and skin disorders are individually related to psychosocial disability, their coexistence may worsen the underlying psychological illness, influencing the management and treatment of these patients. 

Our study provides a broad overview of the available evidence regarding the association between FM and dermatological disorders. 

Despite the potential impact on clinical practice, some limitations need to be recognised. First, the current literature on skin conditions in FM is still scarce, and the available studies are highly heterogeneous in terms of design, sample size, adequate control group, patient demographics (such as sex ratio), and classification criteria. Moreover, a significant proportion of the included studies were cross-sectional studies of moderate quality; hence, other sources of bias may arise when dealing with retrospective data, affecting the generalizability of the results.

## 5. Conclusions

In conclusion, our review suggests that a relationship between FM and cutaneous diseases may exist, although the mechanisms are poorly understood. Comorbid FM may be a contributing factor that worsens skin conditions and lowers overall quality of life. Additionally, since FM represents a significant economic burden to the healthcare system and society, understanding the biopsychosocial causes underlying this possible association could affect not only FM-related costs but also expenses related to skin diseases, leading to more cost-effective treatments. 

For these reasons, a comprehensive approach to dermatological examination is necessary, and it is important to assess if comorbid FM is present so that clinicians can decide how best to manage and treat both conditions and refer patients to a rheumatologist if needed. Future research is necessary to clarify the causal mechanisms underlying the potential correlation between these disorders.

## Figures and Tables

**Table 1:** Quality assessment of the included studies according to the Newcastle-Ottawa Scale (NOS).

Author, Year	Item S1	Item S2	Item S3	Item S4	Item C1	Item E1	Item E2	Item E3	Score
Acar, 2020 [15]	a*	a*	a*	a*	a**	c	a*	d	*******
Askin, 2018 [16]	a*	b	a*	a*	a**	c	a*	d	******
Bruze, 2022 [17]	b	b	a*	a*	a*	a*	a*	d	*****
Dogramaci, 2008 [18]	a*	b	a*	a*	c	c	a*	d	****
Erdogan, 2016 [19]	a*	a*	a*	a*	a**	c	a*	d	*******
Hopkins, 2023 [20]	b	b	b	a*	a*	c	a*	d	***
Khudhair, 2018 [21]	a*	b	a*	a*	a**	c	a*	d	******
Kepekçi, 2018 [22]	a*	b	c	a*	a*	c	a*	d	****
Kridin, 2020 [23]	b*	b	a*	a*	a**	d	a*	d	******
Laniosz, 2014 [24]	b	b	c	c	c	d	c	d	-
Mathkhor, 2020 [25]	a*	b	a*	a*	a**	c	a*	d	******
Mathkhor, 2020 [26]	a*	b	a*	a*	a**	c	a*	d	******
Mathkhor, 2020 [27]	a*	b	a*	a*	a**	c	a*	d	******
Oktayoglu, 2013 [28]	a*	b	a*	a*	c	c	a*	d	****
Oktayoğlu, 2015 [29]	a*	b	a*	a*	a**	c	a*	d	******
Prens, 2022 [30]	b	b	d	C	c	d	c	d	-
Surucu, 2022 [31]	a*	b	a*	a*	a*	c	a*	d	*****
Thune, 2005 [32]	a*	a*	c	c	c	c	c	d	**
Torresani, 2009 [33]	a*	a*	b	a*	c	c	a*	d	****
Yazmalar, 2016 [34]	a*	b	a*	a*	a**	c	a*	d	******
Yener, 2013 [35]	a*	b	a*	a*	a**	c	a*	d	******

Legend: C, Comparability; E, Exposure; S, Selection. A study can be represented by a letter (a,b,c,d) for each numbered item according to the Selection, Comparability, and Exposure categories. The stars obtained by each article are summed up to obtain a final score representing the overall quality of the study, reported in the last column.

**Table 2:** Summary of systematic reviews assessing the association between fibromyalgia and psoriasis.

Author, Year	Country	Enrolment	Recruitment	Design	Patients (% F)	Controls (% F)	FM Criteria	Skin Manifestation
**Psoriasis**								
Kridin, 2020 [23]	Israel	2017	Health Care Database	Cross-sectional	18,598 (91) FM	36,985 (91)	Code	Psoriasis
Mathkhor, 2020 [25]	Iraq	2018–2020	Outpatients and inpatient	Cross-sectional	70 (57)	70 (57)	ACR 1990	Psoriasis
Thune, 2005 [32]	Norway	1997–2000	Consecutive outpatients	Cross-sectional	1269 (56)	NR	ACR 1990	Psoriasis
**Chronic urticaria**								
Mathkhor, 2020 [26]	Iraq	2019–2020	Outpatients	Cross-sectional	82 (57.4)	86 (58.2)	ACR 1990	Chronic urticaria
Torresani, 2009 [33]	Italy	2002–2004	Consecutive outpatients	Cross-sectional	126 (68)	50 (70)	ACR 1990	Chronic urticaria
Oktayoglu, 2013 [28]	Turkey	NA	Outpatients	Cross-sectional	40 (70)	38 (57)	ACR 1990	Chronic urticaria
Yener, 2013 [35]	Turkey	NR	Outpatients	Cross-sectional	72 (65)	67 (70)	FIQ questionnaire	Chronic urticaria
**Contact allergy**								
Bruze, 2022 [17]	Sweden	2017–2018	Fibromyalgia Association	Cross-sectional	119 (100) FM 334 (100) Dermatitis	206 (100)	NR	Contact allergy
Hopkins, 2023 [20]	Sweden	2017–2018	Fibromyalgia Association	Cross-sectional	119 (100) FM	123 (100) Dental 386 (100) Dermatitis	NR	Contact allergy
**Acneiform disorders**								
Acar, 2020 [15]	Turkey	2017–2018	Consecutive outpatients	Cross-sectional	100 (100)	100(100)	ACR 2010	Rosacea
Mathkhor, 2020 [27]	Iraq	2019–2020	Outpatients	Cross-sectional	91 (65)	84 (64)	ACR 1990	Acne vulgaris
Yazmalar, 2016 [34]	Turkey	2014–2015	Outpatients	Cross-sectional	88(68)	76 (81)	ACR 1990	Acne vulgaris
**Hidradenitis suppurativa**								
Prens, 2022 [30]	Nederland	NR	National web survey	Cross-sectional	6156 (62.6)	NR	Self-reported	Hidradenitis Suppurativa
**Vitiligo**								
Askin, 2018 [16]	Turkey	NR	Outpatients	Cross-sectional	35 (48.6)	45 (48.9)	ACR 2010	Vitiligo
Khudhair, 2018 [21]	Iraq	2015–2016	Outpatients	Cross-sectional	100 (54)	200 (63)	2012 Canadian guidelines criteria for FM	Vitiligo
**Miscellaneous**								
Dogramaci, 2008 [18]	Turkey	2007	Outpatients	Cross-sectional	66 (100) FM	79 (100)	ACR 1990	Miscellaneous
Erdogan, 2016 [19]	Turkey	2016	Outpatients	Cross-sectional	105 (100) FM	105 (100)	ACR 2010	Miscellaneous
Kepekçi, 2018 [22]	Turkey	NR	Outpatients	Cross-sectional	120 (50) FM	120 (50)	ACR 2016	Miscellaneous
Laniosz, 2014 [24]	USA	2008	Outpatients	Cross-sectional	845 (90.7) FM	NR	ACR 1990	Miscellaneous
Surucu, 2022 [31]	Turkey	NA	Outpatients	Cross-sectional	77 (89) FM	74 (91)	ACR 2010	Miscellaneous
Oktayoğlu, 2015 [29]	Turkey	NA	Outpatients	Cross-sectional	46 (82) FM	51 (78)	ACR 1990	Pathergy test

Legend: ACR, American College Rheumatology; FIQ, Fibromyalgia Impact Questionnaire; FM, Fibromyalgia; NR, not reported; VASI, vitiligo area scoring index.

## Data Availability

Data are available upon request from the corresponding author.
